# Hospital Uncharity

**Published:** 1893-02-18

**Authors:** 


### HOSPITAL OPINION.

[Contributions critical, suggestive, controversial, and technical are invited for this column, which is open to everyone who has anything to say that is of practical value and interest.]

HOSPITAL UNCHARITY.

A CORRESPONDENT writes: As we look around the out-patient department of any of our great hospitals we are lost in amazement at the number of patients who throng the benches, and still more so at the prosperous appearance of many of those who consider themselves fit objects for charity. But a little experience teaches us that though these people have no objection to receiving the doctor's advice, together with twenty ounces of physic, and a bottle of lotion, without payment, yet most of them would scorn to receive charity in any other form.

The legislature, too, it would seem, recognises this difference of degree in charity, for while the pauper who receives a loaf of bread from the parish is precluded from voting for members of Parliament yet he who receives a bottle of medicine from the parish doctor is not so precluded. Parish relief and hospital relief are in this way differentiated, and he who would reject the one, unhesitatingly embraces the other.

The very perfection of our hospitals may have something to do with this ready acceptance of their aid; the lavishness of their organisation—so different from workhouse methods—prevents the well-clad occupants of the out-patient benches from feeling any qualms.

As a nation we flatter ourselves that our medical charities are a conspicuous example to the world, and that this opinion is not the result of insular prejudice foreigners are always ready to acknowledge. Our hospitals are unsurpassed in all respects, and can always attract the highest medical skill for the benefit of the inmates. Time and talent are ungrudgingly given to the patients, and the nursing is such as "the ladies who ride in their carriages" can hardly command. The charity of the medical profession is so spontaneous that it has almost become a reflex action, and the public are so accustomed to take it as a matter of course that occasionally the line is over-stepped, and injustice done both to the institution and to the profession. Patients present themselves at our hospitals who can in no sense be regarded as objects of charity, thereby defrauding the poor of what is intended solely for them, and imposing on the doctors, who, although they are always ready to attend to the poor gratuitously, naturally object to give money'sworth to those who can afford to pay for what they receive. However simple it may seem theoretically to prevent imposition, it is found practically almost impossible to do so. The patient who comes armed with a subscriber's letter of recommendation may be a fit and proper person to receive charity or he may have obtained the letter by fraud. If the hospital authorities are too particular in making inquiries the subscriber may take offence; moreover, the medical officers have rarely the time to devote to inquiring into the circumstances of every patient. Something might be done to minimize this evil if the members of the lay committee would take it in turns to interview the new patients every morning. This would not take long, and would certainly be more useful work than much that the visitor for the day usually performs.

Another way in which charity is converted into *un*charity is by the acceptance of annual donations from friendly societies as a set-off for services rendered to their members. Charity cannot be bought and sold; if the societies do not pay the full value for what they receive they should recognise that their donation is merely a token of their gratitude. If, on the other hand, the donation is supposed to represent full payment for services rendered, the hospital should refuse it. The proper person to receive it is the medical officer of the society, a reserve fund being established if necessary to pay special fees to a consultant when further advice is required. Anything of the nature of a *quid pro quo* savours too much of trade to be compatible with charity. In this respect the donation boxes in the out-patient departments of our hospitals are to be regarded with suspicion; for a patient who has no right to receive benefits gratuitously often salves his attenuated conscience by ostentatiously depositing therein a very small coin of the realm.

Again, a fruitful source of abuse is the wholesale granting of sick certificates. In some cases there can be no objection to signing them, but those required by the School Board, for instance, certifying that children are unfit for school, should be refused. They are not for the benefit of the parents, and if the School Board requires them they should either appoint a medical inspector or pay the certifying medical attendant; but it is monstrous to expect the profession to work gratuitously for a public department that has the wealth of the nation at its back. It is no part of the duty of a hospital official to give these certificates, and if the plan were adopted of telling patients that they could only be given after a summons had been issued against them it would be sufficient to protect them from any penalty, and would soon compel the School Board to obtain their certificates in a legitimate and honest way.

There is a growing tendency amongst what are called the better classes of seeking admission into the pay wards of a hospital, where for a nominal fee they can have the best treatment and nursing. Some of the London hospitals are great sinners in this respect. If the patients are in reduced circumstances there can be no objection, but it should be recognised that that is the reason why the benefits of the charity are extended to them. People who can well afford to pay the usual fees ought not under any circumstances to be admitted, to have a toe amputated or hæmorrhoids crushed, into a hospital that is supported by charity. An institution where medical relief is bought and sold, whether at stores prices or otherwise, is a doubtful charity at the best, and it has no right to usurp the name and prostitute the traditions that for centuries have been the glory of our hospitals.

